# Feasibility of a randomized hypertension screening initiative in the perioperative setting

**DOI:** 10.1186/s13741-021-00210-7

**Published:** 2021-11-22

**Authors:** Sofia I. Diaz, Luying Yan, Feng Dai, Bin Zhou, Matthew M. Burg, Robert B. Schonberger

**Affiliations:** 1 Department of Anesthesiology, Yale School of Medicine, 333 Cedar Street, TMP 3, New Haven, CT 06520 USA; 2 Yale Center for Analytical Sciences, Yale School of Public Health, 300 George Street, Ste 555, New Haven, CT 06511 USA; 3 Department of Cardiovascular Medicine, Yale School of Medicine, 333 Cedar Street, New Haven, CT 06520 USA

**Keywords:** Preoperative blood pressure screening, Hypertension, Home blood pressure monitoring

## Abstract

**Objectives:**

This study sought to assess feasibility of a randomized trial of blood pressure intervention (home blood pressure monitoring vs. counseling) in the preoperative clinic and the baseline rates of primary care follow-up after such interventions.

**Methods:**

A prospective randomized feasibility study was performed at Yale New Haven Hospital Preadmission Testing Clinic. A sample of 100 adults, with elevated blood pressure, were recruited during their preadmission visit, and randomized 1:1 to receive brief BP counseling and an educational brochure versus additionally receiving a home BP monitor (HBPM) with a mailed report of their home readings. At 60-day post-surgery telephone follow-up, investigators asked whether participants had primary-care follow-up; had new/adjusted hypertension treatment; and felt satisfied with the study.

**Results:**

There were 51 patients in the counseling group and 49 in the HBPM group. Of 46 patients in the HBPM group who returned their monitors, 36 (78%) were hypertensive at home. At 60 days post-surgery, 31 (61%) patients in the counseling group and 30 (61%) in the HBPM group were reached by telephone with the remaining followed by EHR. Thirty-six (71%) patients in the counseling group and 36 (73%) in the HBPM group had seen their primary care provider. Seventeen of 36 (47%) in the counseling group and 18 of 31 (58%) in the HBPM group received new or adjusted hypertension medications. Sixty-one participants answered questions regarding their satisfaction with the study with 52 (85%) reporting that they felt moderately to very satisfied.

**Conclusions:**

This feasibility study suggests that interventional blood pressure trials in the preoperative clinic are feasible, but telephone follow-up leads to significant gaps in outcome ascertainment.

**Trial registration:**

Clinicaltrials.gov, NCT03634813. Registered 16 of August 2018.

## Introduction

Poorly controlled hypertension increases risk for cardiovascular disease (CVD) (Grimm Jr. et al. 1985; Stamler et al. 1989; Xu et al. 2015) and, remains the leading modifiable cause of death and disability-adjusted life years worldwide (Lim et al. 2012). Despite continued efforts to address undertreated blood pressure elevation, approximately 22% of Americans with high blood pressure are unaware of the condition, and 32% are not taking anti-hypertensive medications (Ostchega et al. 2008).

Several investigations have attempted to address this public health opportunity through hypertension screening outside of the traditional setting of the primary care office, including in both the acute care setting (Tanabe et al. 2008) and in the general community with notable success (Victor et al. 2018).

Within the field of anesthesiology, the Merit-Based Incentive Program (MIPS) included hypertension screening in its 2007 anesthesiology specific quality metrics, and subsequent literature established plausible thresholds at which preoperative blood pressures appeared reasonably predictive of longitudinal blood pressure elevation (Schonberger et al. 2012; Schonberger et al. 2015; Schonberger et al. 2018). Nevertheless, while isolated teams have attempted to bring hypertension screening and treatment into perioperative workflows (Pfister et al. 2020), randomized interventional studies based on these efforts have remained unexplored.

In the effort to assess the feasibility of a randomized blood pressure intervention in the preoperative period and to understand baseline rates of blood pressure follow-up following such intervention, we pursued a randomized feasibility trial of blood pressure screening, education, and follow-up with vs. without longitudinal home blood pressure measurement in a cohort of 100 patients who presented to our preoperative testing center with blood pressures in the hypertensive range. The endpoints of interest included metrics to assess feasibility to execute the protocol including participants’ ability to complete home blood pressure monitoring and the ability to reach participants for follow-up via telephone and EHR tracking. Secondly, we measured the observed prevalence of post-operative primary care follow-up and patient perspectives on the study protocol, to better enable appropriate powering of a future trial targeting outcomes of improved blood pressure treatment.

## Methods

### Inclusion and exclusion

After approval by the Yale School of Medicine Institutional Review Board and registration on Clinicaltrials.gov (NCT03634813), a sample of 100 patients scheduled for surgery was recruited during their preadmission visit at Yale-New Haven Hospital between November of 2018 and November of 2019. Inclusion criteria included age > 18 years, the ability to understand English, and a clinic-measured blood pressure of at least 140 mmHg systolic or 90 mmHg diastolic calculated as the mean of two successive measurements 5 min apart. These blood pressures were measured using an automated oscillometric blood pressure device rather than manually by clinicians in accordance with literature demonstrating the superior reliability of such measurements for hypertension screening (Roerecke et al. 2019). Participants who were unable or unwilling to independently operate a brachial artery home blood pressure monitor, those who could not give proper consent, or those for whom the date of surgery was within 48 h of the preoperative visit, were excluded.

### Randomization

After informed consent was obtained, all participants were given a questionnaire that included measures of medication adherence previously validated on hypertensive patients (Voils et al. 2012). Participants were then randomly assigned to one of two groups in a 1:1 fashion stratified by gender. The two groups were defined as follows: One group received brief counseling regarding their apparent hypertension and the importance of blood pressure follow-up. They then received a follow-up letter and an educational pamphlet by mail published by the National Heart, Lung, and Blood Institute entitled “Your guide on lowering blood pressure” (National Heart, Lung, and Blood Institute; National Institutes of Health; US Department of Health and Human Services 2018), which includes advice regarding diet, exercise, and lifestyle changes that can be implemented to improve blood pressure control. The second group received the educational pamphlet at the time of enrollment and was also fitted with a validated Omron MX3 model BP742 home blood pressure monitor (Omron, Shaumberg, IL) (Coleman et al. 2005) and instructed in its use. The HBPM stores timestamped results for up to 50 blood pressure measurements. Participants were sent home with the device and instructed to conduct resting measurements in the morning and evening of each day until the day of surgery and then to bring the device back to the investigators on the day of surgery. Since specific cut-offs for the interpretation of home blood pressure are controversial (Myers et al. 2011), and per the 2017 American Heart Association guidelines, should vary based on cardiovascular disease risk assessment (Whelton et al. 2018), in the present trial, we used mean systolic home BP ≥ 135 mmHg or mean diastolic home BP ≥ 85 mmHg as our HBPM cutoffs. A similar HBPM cutoff of 137/84 mmHg has previously been associated with a 10% increase in the adjusted relative hazard of mortality relative to ideal blood pressure (Tsuji et al. 1997). HBPM participants received a summary report of their mean home blood pressure readings by mail. Unless patients demonstrated sustained normotension on HBPM readings, these letters encouraged follow-up to discuss hypertension management with participants’ primary care physician.

### Outcome assessment

**Fig. 1:**
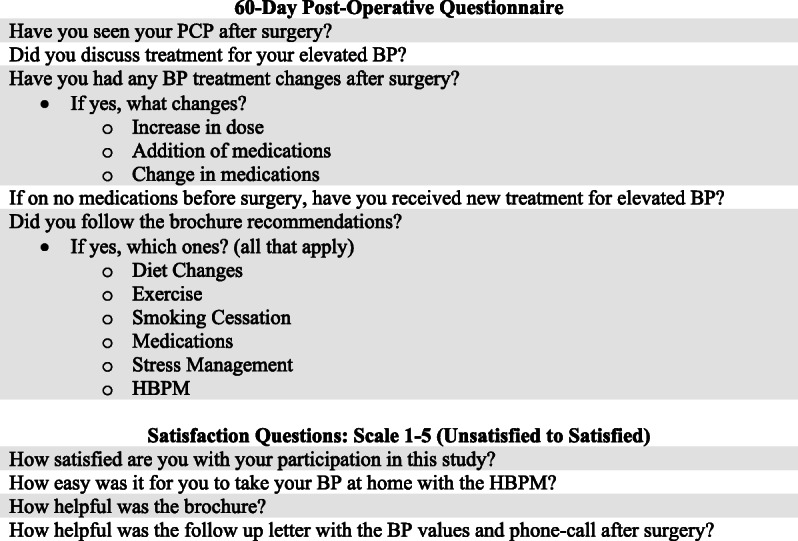
Post-operative questionnaire. PCP, primary care physician; BP, blood pressure; HBPM, home blood pressure monitoring

Sixty-day post-surgery, investigators attempted to reach participants via follow-up telephone call to (a) determine whether the participant had been seen in primary-care follow-up; (b) determine whether the participant had received new or adjusted hypertension treatment, and (c) assess participants’ satisfaction with the study interventions (see Fig. 1 for the list of telephone questions). For those participants who were not reached after three phone call attempts, investigators consulted the electronic health record to look for new or adjusted hypertension treatment.

## Results

### Cohort characteristics

A total of 100 participants were successfully enrolled. The mean (SD) age was 64.9 (11.7). Forty-nine participants were assigned to the HBPM group vs. 51 in the counseling group. Fifty-four (54%) were female and forty-six (46%) male. Seventy-two (76%) participants identified as white, 16 (17%) as Black or African American, and ten (10%) as Hispanic (see Table 1 for a complete demographic description of the cohort stratified by randomized group).

**Table 1:** Demographics

	*Counseling* (*n =* 51)	*HBPM* (*N =* 49)	*Total* (*N =* 100)
*Female*	27 (53%)	27 (55%)	54 (54%)
*Male*	24 (47%)	22 (45%)	46 (46%)
*Hispanic or Latino*	7 (14%)	3 (6%)	10 (10%)
*Asian*	1 (2%)	0 (0%)	1 (1%)
*Black/African American*	11 (22%)	5 (11%)	16 (16%)
*White/Caucasian*	33 (66%)	39 (87%)	72 (72%)
*Other*	5 (10%)	1 (2%)	6 (6%)
*N/A*	1 (2%)	4 (8%)	5 (5%)

Blood pressures in the preadmission testing clinic were 154.22 (±13.05) mmHg/88.88 mmHg (±10.03) in the counseling group, and for the HBPM group 158.10 mmHg (±13.46)/90.25 mmHg (±11.15). Comparing the counseling group vs. the HBPM group, 50 (98%) and 47 (96%) participants had an existing primary care doctor, respectively; 43 (84%) and 42 (85%) had seen their PCP in the last 6 months (of which 36 and 29 saw their PCP in the last 3 months), and 44 (86%) vs. 40 (82%) were aware of a prior diagnosis of hypertension, respectively. The enrolled cohort in our clinic demonstrated higher baseline treatment as compared to prior literature (Ostchega et al. 2008) with 78% and 80% of each group, respectively, reporting that they were prescribed medications to control their blood pressure. Regarding medication adherence, it was similar among both groups. Out of 79 patients among both groups being treated for elevated blood pressure, a total of 64 (81%) agreed or strongly agreed with the statement that they took all their anti-hypertensive medications as prescribed (see Table 2 for more information regarding their hypertensive history).

**Table 2:** Hypertension history

	*Counseling*	*HBPM*	*Total*
*Diagnosis of hypertension?*	***n*** **= 51**	***n*** **= 49**	***n*** **= 100**
*Yes*	44 (86%)	40 (82%)	84 (84%)
*No*	7 (14%)	7 (14%)	14 (14%)
*No answer*		2 (4%)	2 (2%)
*Time since diagnosis*	***n*** **= 43**	***n*** **= 42**^a^	***n*** **= 85**
*0-6 months*	12 (28%)	7 (17%)	19 (22%)
*6-12 months*	3 (7%)	1 (2%)	4 (5%)
*> 12 months*	28 (65%)	34 (81%)	62 (73%)
*No answer*	8	7	15
*Have you been to the Emergency Department due to elevated blood pressure?*	***n*** **= 51**	***n*** **= 49**	***n*** **= 100**
*Yes*	8 (16%)	6 (12%)	14 (14%)
*Are you being treated for hypertension?*	***n*** **= 51**	***n*** **= 49**	***n*** **= 100**
*Yes*	40 (78%)	39 (80%)	79 (79%)
*What type of treatment?*^b^	***n*** **= 39**	***n*** **= 39**	***n*** **= 78**
*Diet changes*	11 (28%)	12 (31%)	23 (29%)
*Exercise*	17 (44%)	13 (33%)	30 (38%)
*Smoking cessation*	5 (13%)	1 (3%)	6 (8%)
*Medications*	35 (90%)	36 (92%)	71 (91%)
*Stress management*	3 (8%)	3 (8%)	6 (8%)
*HBPM*	19 (49%)	8 (21%)	27 (35%)
*No answer*	12	10	22
*If taking medications, how many do you take?*	***n*** **= 38**	***n*** **= 33**	***n*** **= 71**
*1*	15 (39%)	13 (39%)	28 (39%)
*2*	13 (34%)	13 (39%)	26 (37%)
*= or > 3*	10 (26%)	7 (21%)	17 (24%)
*No answer*	13	16	29

*N* equals available answers from questionnaires

^a^2 patients in the HBPM group responded to this question, despite not answering positively to a diagnosis of hypertension

^b^Multiple answers were permitted; hence, percentages do not add to 100%

When examining hypertension treatment by gender, a similar proportion of the female participants (accounting for 54% of the sample) had a PCP (98% vs. 95%) and received hypertension treatment (93% vs. 95%) as their male counterparts. However, the female participants were less likely to have a prior diagnosis of hypertension (78% vs. 91%) or to be compliant with treatment (74% vs. 88%), compared to male participants. Regarding race, Caucasian participants accounted for 72% of the sample population while non-Caucasian participants accounted for 28% of the sample population. A similar proportion of Caucasian and non-Caucasian participants had a PCP (99% vs. 93%) and a prior diagnosis of hypertension (83% vs. 86%), was receiving treatment for hypertension (93% vs. 96%), and reported being compliant with their medications (89% vs. 87%). Delving deeper into the non-Caucasian group, 16 (57%) were Black/African-American; among these Black participants, 100% had a PCP, 94% had a prior diagnosis of hypertension, 93% were receiving treatment for hypertension, and 100% reported being adherent with their medications.

### Protocol completion and feasibility

**Fig. 2:**
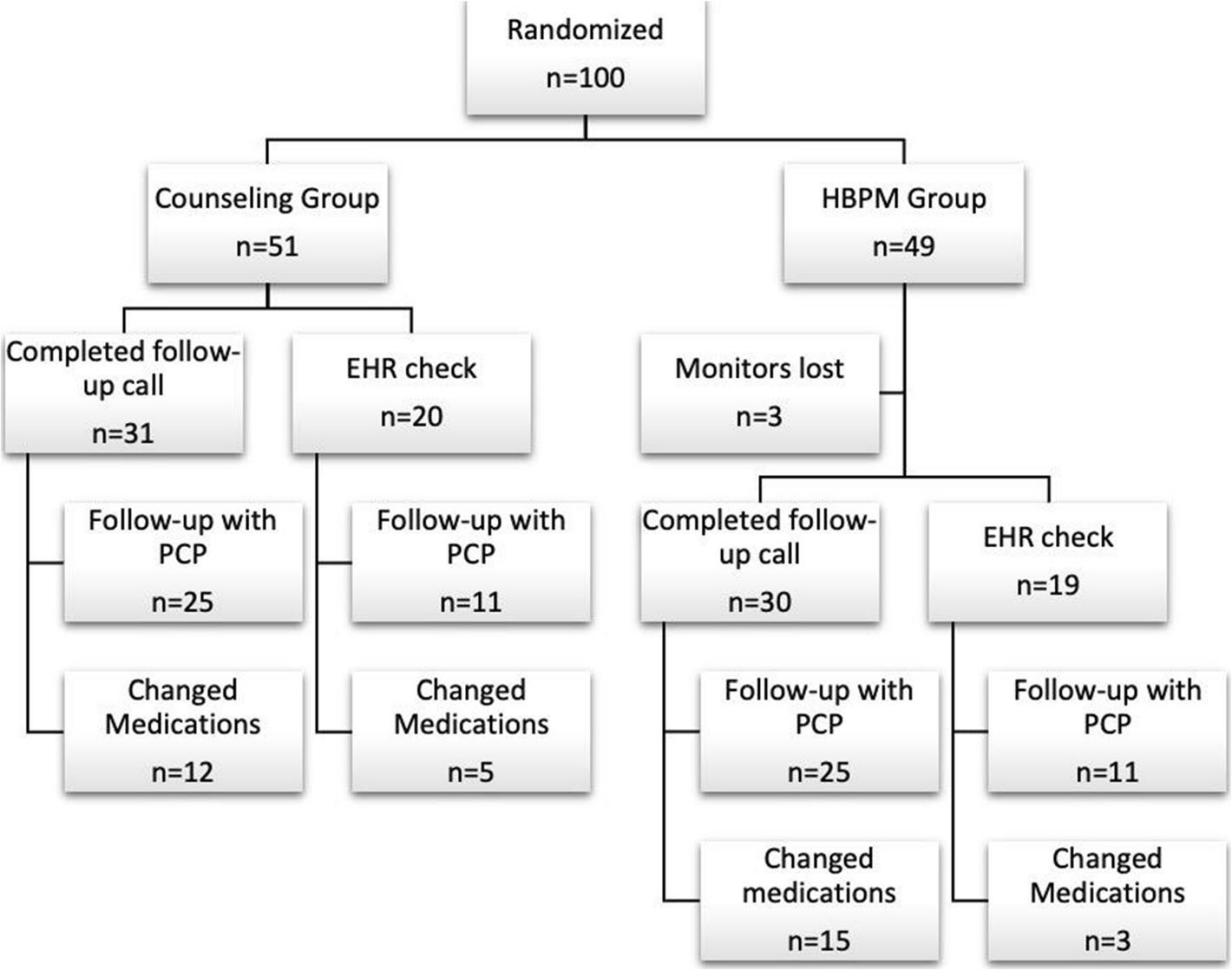
Flow chart depicting allocation of randomized participants and main results. HBPM, home blood pressure monitor; PCP, primary care physician; EHR, electronic health record

Out of 100 participants enrolled, 31 out of 51 (61%) patients in the counseling group and 30 out of 49 (61%) in the HBPM group were able to be reached for a follow-up questionnaire. For the 39 participants who could not be contacted, a chart review in the EMR was performed to identify which patients had seen their PCP and had a change in their medication regime. If no information was found, they were coded as missing information (see Fig. 2).

**Fig. 3:**
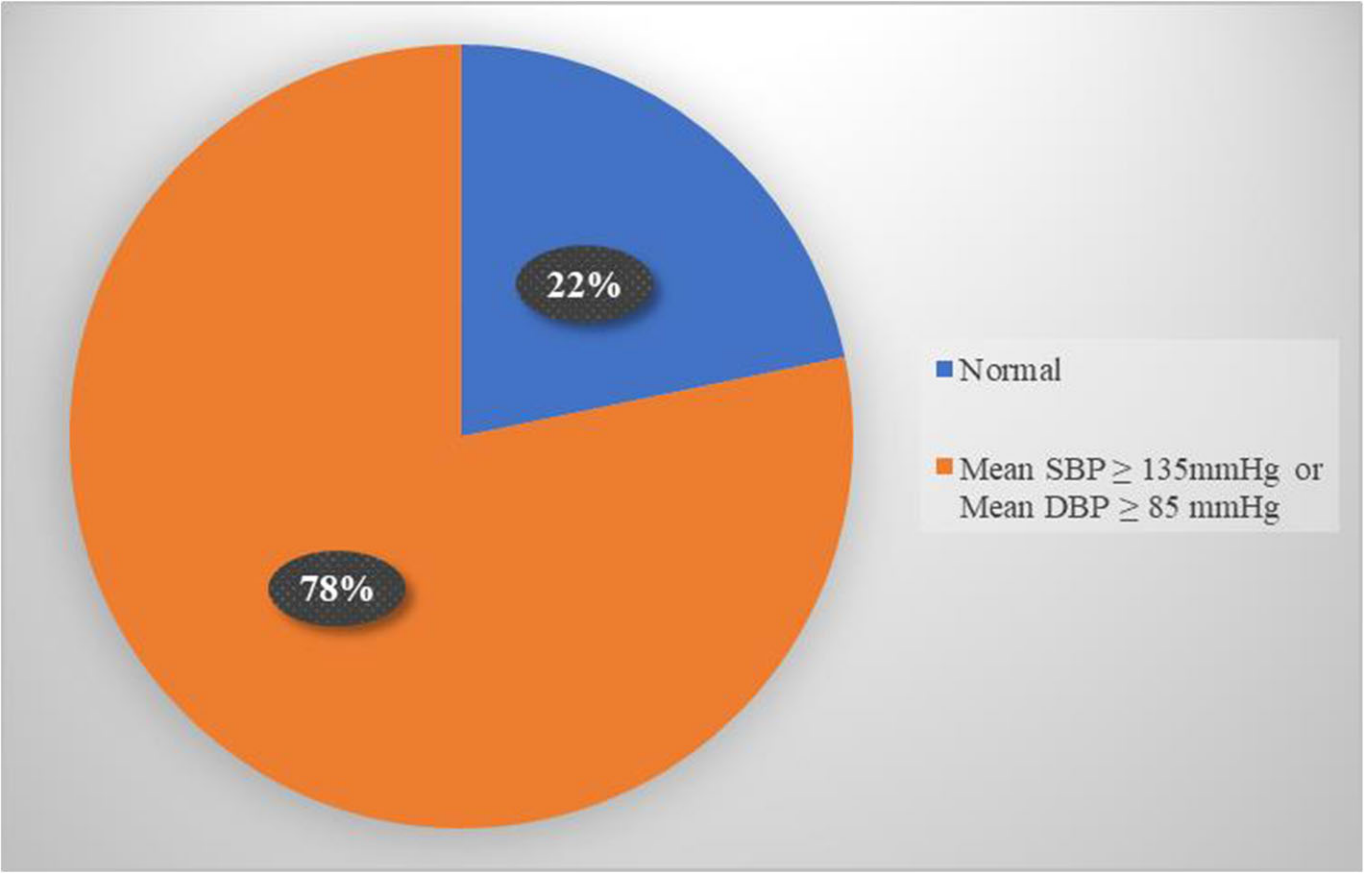
Home blood pressure monitor cutoffs. Cutoff used was mean systolic HBP ≥ 135 mmHg or mean diastolic HBP ≥ 85 mmHg. HBP, home blood pressure; SBP, systolic blood pressure; DBP, diastolic blood pressure

Of the forty-nine home blood pressure monitors provided, 46 (94%) were returned, consistent with our prior experience of adherence to such monitoring among study participants in the preoperative population (Schonberger et al. 2018). The median number of blood pressure readings among these participants was 26 (IQR = 16-46). Thirty-six (78%) of the participants that returned the HBPM had elevated home BP, as defined by our a priori cut off of a mean systolic HBP ≥ 135 mmHg or mean diastolic HBP ≥ 85 mmHg (Myers et al. 2011; Whelton et al. 2018; Tsuji et al. 1997) (see Fig. 3).

### Primary outcomes

Primary care follow-up 60 days after surgery was completed by 36 (71%) patients in the counseling group and 36 (73%) patients in the HBPM group. Of the 72 patients that visited their primary care physician post-operatively, 29 out of 36 (81%) in the counseling group reported discussing hypertension treatment with their PCP vs. 32 out of 36 (89%) in the HBPM group. Regarding outcomes by gender, males were more likely to follow-up with their PCP (80% vs. 65%) than females. Between Caucasians and non-Caucasians, the follow-up rate was similar (71% vs. 75%).

**Fig. 4:**
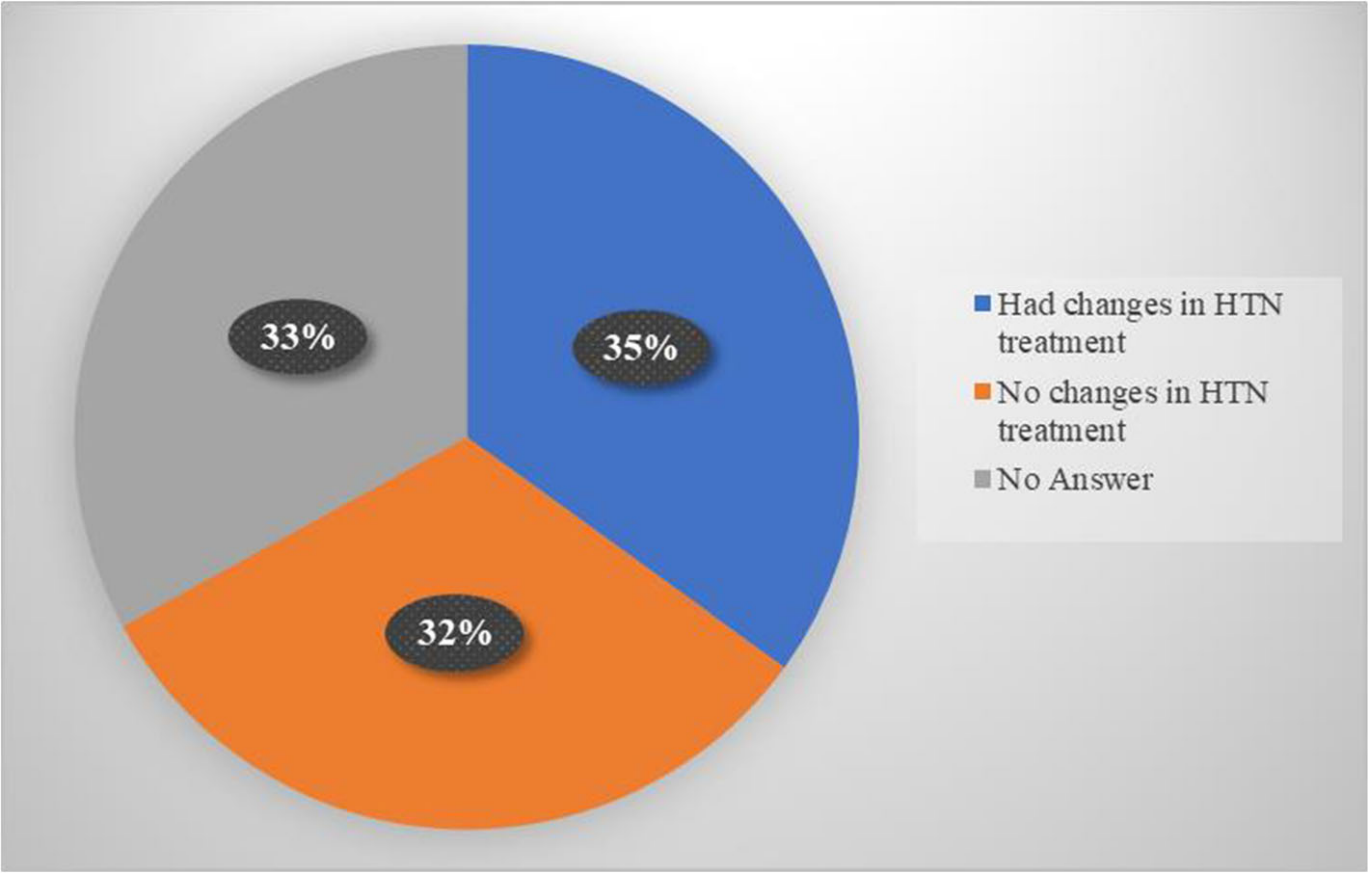
Percentage of patients with changes in their blood pressure treatment at 60 days of follow-up. Both the counseling and HBPM group had an even distribution regarding treatment. HTN, hypertension; HBPM, home blood pressure monitoring group

Modification of hypertensive treatment took place in 17 out of 36 (47%) patients in the counseling group and 18 out of 31 (58%) in the HBPM group that answered this question (Fig. 4). The changes in the counseling group included 4 (24%) patients with an increase in dosing, 7 (41%) patients who had a change in medications prescribed, and 9 (53%) patients that had medications added. In the HBPM group, these numbers were 6 (33%), 5 (28%), and 8 (44%), respectively. Out of the patients that were not on medications pre-operatively, 3 out of 13 (23%) in the control group and 3 out of 16 (19%) in the intervention group were started on medications. By gender, males were more likely to have a change in their medication (54% vs. 19%) than females. By race, non-Caucasians were similarly likely to have a change in their medications as Caucasians (39% vs. 33%).

### Secondary outcomes and participant acceptance of the interventions

Out of the 61 participants who we were able to reach for post-surgical follow-up, 30 belonged to the HBPM group and 31 to the counseling group. They were all asked several questions regarding satisfaction with the protocol participation, as listed in Fig. 1. Out of the 61 participants, 33 were female (54%) and 28 were male (46%). Forty-eight (79%) were Caucasian and 13 (21%) were non-Caucasian. Further subdivision of the non-Caucasian group revealed that 9 were Black/African American (69%), 1 was Asian (8%), and 3 were non-Caucasian Hispanic (23%).

When asked if they remembered the NIH brochure and had put in practice any recommendations, 43 out of sixty-one (70%) of the participants answered that they did. The most common interventions cited by participants were diet changes and exercise, endorsed equally by 61% of participants (see Table 3). However, it is important to note that this number does not necessarily reflect the patients who were newly adherent to the brochure, since some participants were already implementing certain strategies before obtaining the brochure information. Of the 43 participants who reported recalling the brochure recommendations, 24 (40%) were female and 19 (31%) male. Seventy-three percent of Caucasian participants and 62% of non-Caucasian participants reported recollection of the brochure recommendations.

**Table 3:** Methods to lowering blood pressure implemented from the NIH brochure

	*Counseling*	*HBPM*	*Total*
*Changes adopted from NIH brochure*	***n*** **= 24**	***n*** **= 32**	***n*** **= 56**
*Diet changes*	17 (71%)	17 (53%)	34 (61%)
*Exercise*	17 (71%)	17 (53%)	34 (61%)
*Smoking cessation*	2 (8%)	2 (6%)	4 (7%)
*Medications*	11 (46%)	10 (31%)	21 (38%)
*Stress management*	3 (5%)	5 (8%)	8 (7%)
*Home blood pressure monitor—HBPM*	9 (13%)	11 (34%)	20 (36%)
*No answer*	27	17	44

Multiple answers per patient allowed; therefore, columns do not add to 100%. *N* equals available answers from patients reached by phone, as well as chart reviews

**Fig. 5:**
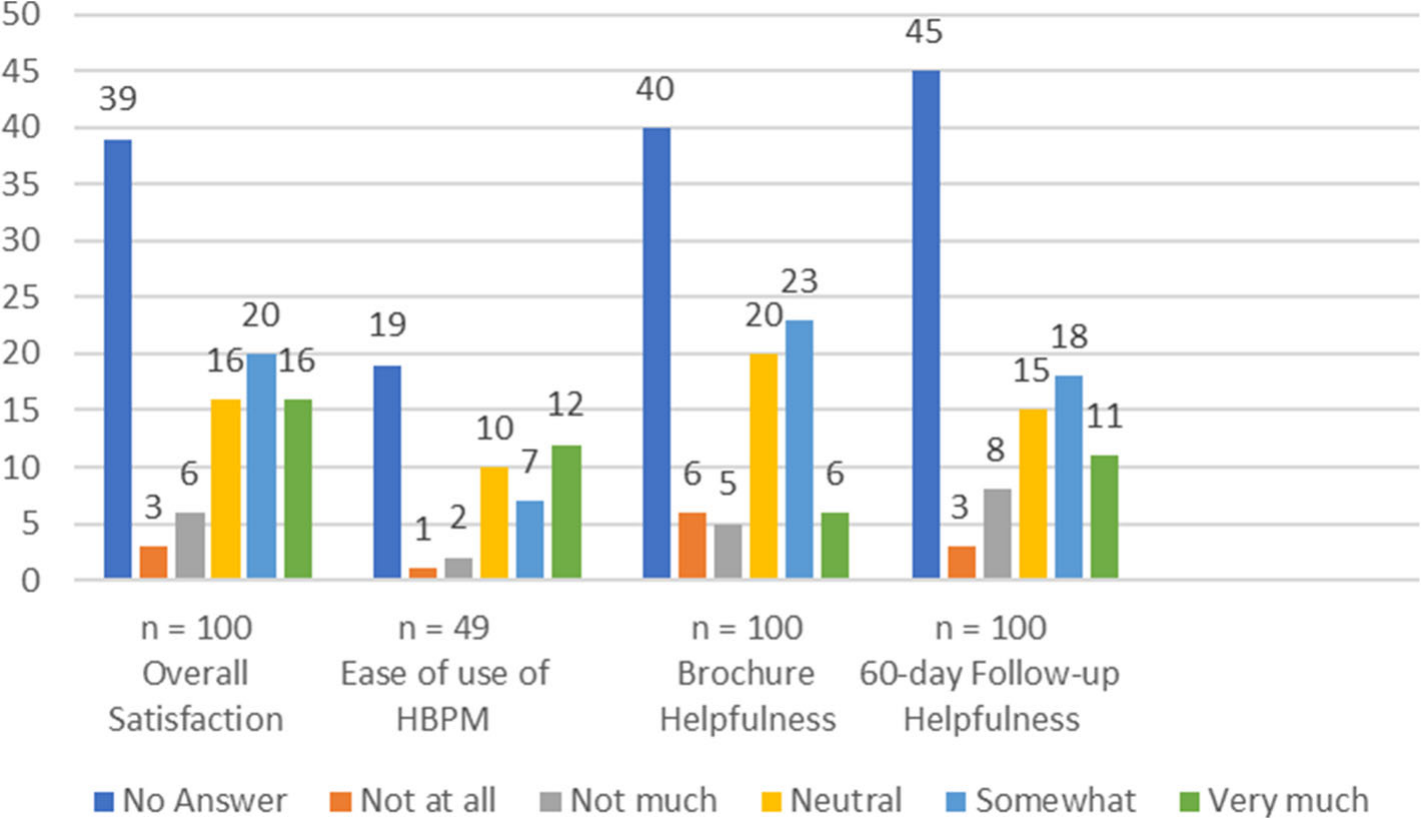
60-day follow-up satisfaction questionnaire. Satisfaction scores measured with a 5-point Likert scale. Values given in number of patients that answered each individual question. HBPM, home blood pressure monitor

Regarding protocol acceptance, participants were asked to rate their satisfaction with the study on several 5-point Likert scales ranging from 1 “not at all” through 5 “very much.” Overall, fifty-two out of 61 (85%) participants, who answered, were neutral to very satisfied with their participation in the study. Forty-nine out of 60 (82%) patients thought the NIH brochure was neutral to very helpful, and when asked about the follow-up call, 44 out of 55 (80%) patients that answered ranked it as neutrally helpful to very helpful. Of the patients in the HBPM group, 28 out of 30 (93%) that were reached found it neutral to very easy to utilize, although a total of 7 out of the 46 (15.2%) patients who returned the HBPM expressed that adding the HBPM peri-operatively was an added stressor (see Fig. 5).

## Discussion

Elevated blood pressure is a common, and modifiable cardiovascular disease (CVD) risk factor in patients presenting for surgery. Programs, such as the Perioperative Enhancement Team (POET) at Duke University, have created multidisciplinary teams with the intention of reducing perioperative risk factors by sending their patients to subspecialized clinics for optimization with good results (Aronson 2016; Aronson et al. 2018; Schonberger 2016). However, interventions focused on care coordination for newly diagnosed or uncontrolled hypertension have been more limited (Pfister et al. 2020). The present feasibility study demonstrates that preoperative clinic blood pressure interventions are feasible and accepted by most participants, but their impact on blood pressure management is not established.

Several important results of this study deserve highlighting. First, while HBPM monitoring was successfully implemented in 94% of participants, it is not clear whether the marginal value gained from the relatively time-intensive process of instructing patients on HBPM use and tracking HBPM return carries sufficient pay-off over and above the simple intervention of a brief counseling session at the time of preoperative evaluation. Although HBPM has been shown to have a higher sensitivity and specificity for truly elevated blood pressure as compared to office measurements (Bosworth et al. 2011; Cahan et al. 2011; Mallick et al. 2009), it is notable that almost 4 in 5 participants who were deemed to be hypertensive in the preoperative clinic indeed had the diagnosis confirmed in their home blood pressure readings. It is not clear whether the marginal improvement in diagnostic fidelity found in HBPM outweighs the practical benefits of a more widely acceptable brief counseling intervention for hypertensive presurgical patients.

Second, it is notable that the study cohort demonstrated significant changes in hypertension treatment at 60 days follow-up regardless of group assignment. The fact that 35 out of 100 patients in the trial had a change in hypertensive medication treatment at 60 days of follow-up may lend credence to the potential importance of preoperative counseling to encourage such follow-up.

Third, although we did not formally assess the ease of recruiting participants, we did observe that there were several challenges in the recruitment process that could be addressed in a future study iteration. Study recruiters depended on the providers in the pre-admission testing clinic to keep patients after their visit, and this step led to many potential participants leaving before recruitment was attempted. We believe that if all phases of screening and enrollment could be done by one provider, it would increase rapport and facilitate successful entry into the study. In addition, several potential participants were happy to measure home blood pressures but were unwilling to be randomized within the study, a factor that is commonly encountered in many randomized trials.

Of additional importance, the finding that a large percentage of the overall cohort were not able to be reached by telephone at 60 days suggests that future pragmatic trials of larger scale blood pressure screening interventions in the perioperative period may benefit from greater reliance on Electronic Health Record (EHR) follow-up to ascertain blood pressure treatment changes. While this would, of course, limit potential follow-up to patients within integrated EHR systems, the alternative of telephone follow-up itself carried with it a 39% failure to follow-up. As integrated electronic health records gain market penetrance, we suggest that the benefits of EHR follow-up are likely to increase.

A further limitation that is important to note is that the present study should be interpreted cautiously before generalizing to other contexts, as our sample population showed notably high PCP utilization (> 95%) as compared to that exhibited in the general US population (Levine et al. 2019). This finding likely reflects that the preoperative clinic where recruitment took place primarily served patients who were outpatients preparing for significant surgical interventions, and for such a population, it is not surprising that they would be engaged in outpatient healthcare beyond the rates seen in the general population. It may be that a similar intervention, focused on patients with lower baseline primary care engagement would be more impactful. Similarly, the rates of medication adherence in our population were far higher than would be expected in the general US population. It is possible that self-reported medication adherence was skewed higher in the context of anxious patients who were eager to prepare themselves for surgical interventions.

Another notable limitation is that, while we excluded non-English speakers, we did not assess primary language. It is possible that some patients may have had language barriers that were not addressed in our enrollment questionnaire and that may have impacted the feasibility of the intervention in some participants.

In conclusion, our feasibility study suggests that preoperative clinic blood pressure screening and intervention is feasible and accepted by patients. Given the accuracy of preoperative clinic blood pressure measurement for predicting longitudinally elevated blood pressures, it remains unestablished whether additional diagnostic accuracy obtained from home blood pressure monitoring in the preoperative period is worthwhile. Future studies are required to delineate whether preoperative hypertension screening programs can have potentially long-term benefits for cardiovascular health among patients presenting for surgery.

## Data Availability

The datasets used and/or analyzed during the current study are available from the corresponding author on reasonable request.

## Abbreviations

CVD: Cardiovascular disease
EHR: Electronic health record
HBP: Home BP
HBPM: Home blood pressure monitor
MIPS: Merit-Based Incentive Program
NHLBI: National Heart, Lung, and Blood Institute
NIH: National Institutes of Health
PCP: Primary care physician
POET: Perioperative enhancement team

## References

[CR1] Aronson S (2016). Moving toward a cardiovascular perioperative enhancement team. J Cardiothorac Vasc Anesth.

[CR2] Aronson S, Westover J, Guinn N, Setji T, Wischmeyer P, Gulur P, Hopkins T, Seyler TM, Lagoo-Deendayalan S, Heflin MT, Thompson A, Swaminathan M, Flanagan E (2018). A perioperative medicine model for population health: an integrated approach for an evolving clinical science. Anesth Analg.

[CR3] Bosworth HB, Powers BJ, Olsen MK, McCant F, Grubber J, Smith V, Gentry PW, Rose C, van Houtven C, Wang V, Goldstein MK, Oddone EZ (2011). Home blood pressure management and improved blood pressure control: results from a randomized controlled trial. Arch Intern Med.

[CR4] Cahan A, Ben-Dov IZ, Mekler J, Bursztyn M (2011). The role of blood pressure variability in misdiagnosed clinic hypertension. Hypertension Res.

[CR5] Coleman A, Freeman P, Steel S, Shennan A (2005). Validation of the Omron MX3 Plus oscillometric blood pressure monitoring device according to the European Society of Hypertension international protocol. Blood Pressure Monit.

[CR6] Grimm RH, Cohen JD, Smith WM, Falvo-Gerard L, Neaton JD (1985). Hypertension management in the Multiple Risk Factor Intervention Trial (MRFIT). Six-year intervention results for men in special intervention and usual care groups. Arch Intern Med.

[CR7] Levine DM, Landon BE, Linder JA (2019). Quality and experience of outpatient care in the United States for adults with or without primary care. JAMA Intern Med.

[CR8] Lim SS, Vos T, Flaxman AD, Danaei G, Shibuya K, Adair-Rohani H, AlMazroa MA, Amann M, Anderson HR, Andrews KG, Aryee M, Atkinson C, Bacchus LJ, Bahalim AN, Balakrishnan K, Balmes J, Barker-Collo S, Baxter A, Bell ML, Blore JD, Blyth F, Bonner C, Borges G, Bourne R, Boussinesq M, Brauer M, Brooks P, Bruce NG, Brunekreef B, Bryan-Hancock C, Bucello C, Buchbinder R, Bull F, Burnett RT, Byers TE, Calabria B, Carapetis J, Carnahan E, Chafe Z, Charlson F, Chen H, Chen JS, Cheng ATA, Child JC, Cohen A, Colson KE, Cowie BC, Darby S, Darling S, Davis A, Degenhardt L, Dentener F, Des Jarlais DC, Devries K, Dherani M, Ding EL, Dorsey ER, Driscoll T, Edmond K, Ali SE, Engell RE, Erwin PJ, Fahimi S, Falder G, Farzadfar F, Ferrari A, Finucane MM, Flaxman S, Fowkes FGR, Freedman G, Freeman MK, Gakidou E, Ghosh S, Giovannucci E, Gmel G, Graham K, Grainger R, Grant B, Gunnell D, Gutierrez HR, Hall W, Hoek HW, Hogan A, Hosgood HD, Hoy D, Hu H, Hubbell BJ, Hutchings SJ, Ibeanusi SE, Jacklyn GL, Jasrasaria R, Jonas JB, Kan H, Kanis JA, Kassebaum N, Kawakami N, Khang YH, Khatibzadeh S, Khoo JP, Kok C, Laden F, Lalloo R, Lan Q, Lathlean T, Leasher JL, Leigh J, Li Y, Lin JK, Lipshultz SE, London S, Lozano R, Lu Y, Mak J, Malekzadeh R, Mallinger L, Marcenes W, March L, Marks R, Martin R, McGale P, McGrath J, Mehta S, Memish ZA, Mensah GA, Merriman TR, Micha R, Michaud C, Mishra V, Hanafiah KM, Mokdad AA, Morawska L, Mozaffarian D, Murphy T, Naghavi M, Neal B, Nelson PK, Nolla JM, Norman R, Olives C, Omer SB, Orchard J, Osborne R, Ostro B, Page A, Pandey KD, Parry CDH, Passmore E, Patra J, Pearce N, Pelizzari PM, Petzold M, Phillips MR, Pope D, Pope CA, Powles J, Rao M, Razavi H, Rehfuess EA, Rehm JT, Ritz B, Rivara FP, Roberts T, Robinson C, Rodriguez-Portales JA, Romieu I, Room R, Rosenfeld LC, Roy A, Rushton L, Salomon JA, Sampson U, Sanchez-Riera L, Sanman E, Sapkota A, Seedat S, Shi P, Shield K, Shivakoti R, Singh GM, Sleet DA, Smith E, Smith KR, Stapelberg NJC, Steenland K, Stöckl H, Stovner LJ, Straif K, Straney L, Thurston GD, Tran JH, van Dingenen R, van Donkelaar A, Veerman JL, Vijayakumar L, Weintraub R, Weissman MM, White RA, Whiteford H, Wiersma ST, Wilkinson JD, Williams HC, Williams W, Wilson N, Woolf AD, Yip P, Zielinski JM, Lopez AD, Murray CJL, Ezzati M (2012). A comparative risk assessment of burden of disease and injury attributable to 67 risk factors and risk factor clusters in 21 regions, 1990–2010: a systematic analysis for the Global Burden of Disease Study 2010. Lancet.

[CR9] Mallick S, Kanthety R, Rahman M (2009). Home blood pressure monitoring in clinical practice: a review. Am J Med.

[CR10] Myers MG, Godwin M, Dawes M, Kiss A, Tobe SW, Grant FC, Kaczorowski J (2011). Conventional versus automated measurement of blood pressure in primary care patients with systolic hypertension: randomised parallel design controlled trial. BMJ.

[CR11] National Heart, Lung, and Blood Institute; National Institutes of Health; US Department of Health and Human Services; “Your Guide to Lowering Blood Pressure”(url: https://www.nhlbi.nih.gov/files/docs/public/heart/hbp_low.pdf as Accessed Nov 2018).

[CR12] Ostchega Y, Yoon S, Hughes J, Louis T (2008). Hypertension awareness, treatment, and control - continued disparities in adults: United States 2005-2006. Services USDoHaH, editor: Centers for Disease Control and Prevention; National Center for Health Statistics.

[CR13] Pfister C-L, Govender S, Dyer RA (2020). A multicenter, cross-sectional quality improvement project: the perioperative implementation of a hypertension protocol by anesthesiologists. Anesth Analg.

[CR14] Roerecke M, Kaczorowski J, Myers MG. Comparing automated office blood pressure readings with other methods of blood pressure measurement for identifying patients with possible hypertension: a systematic review and meta-analysis. JAMA Intern Med. 2019;179(3):351–62. 10.1001/jamainternmed.2018.6551. 10.1001/jamainternmed.2018.6551 PMC6439707 30715088

[CR15] Schonberger RB (2016). Rebranding the perioperative surgical home: lessons from the Duke experience. J Cardiothorac Vasc Anesth.

[CR16] Schonberger RB, Burg MM, Holt NF, Lukens CL, Dai F, Brandt C (2012). The relationship between day-of-surgery and primary care blood pressure among Veterans presenting from home for surgery. Is there evidence for anesthesiologist-initiated blood pressure referral?. Anesth Analg.

[CR17] Schonberger RB, Dai F, Brandt CA, Burg MM (2015). Balancing model performance and simplicity to predict postoperative primary care blood pressure elevation. Anesth Analg.

[CR18] Schonberger RB, Nwozuzu A, Zafar J, Chen E, Kigwana S, Monteiro MM, Charchaflieh J, Sophanphattana S, Dai F, Burg MM (2018). Elevated preoperative blood pressures in adult surgical patients are highly predictive of elevated home blood pressures. J Am Soc Hypertens.

[CR19] Stamler J, Neaton JD, Wentworth DN (1989). Blood pressure (systolic and diastolic) and risk of fatal coronary heart disease. Hypertension.

[CR20] Tanabe P, Persell SD, Adams JG, McCormick JC, Martinovich Z, Baker DW (2008). Increased blood pressure in the emergency department: pain, anxiety, or undiagnosed hypertension?. Ann Emerg Med.

[CR21] Tsuji I, Imai Y, Nagai K (1997). Proposal of reference values for home blood pressure measurement: prognostic criteria based on a prospective observation of the general population in Ohasama, Japan. Am J Hypertension.

[CR22] Victor RG, Lynch K, Li N, Blyler C, Muhammad E, Handler J, Brettler J, Rashid M, Hsu B, Foxx-Drew D, Moy N, Reid AE, Elashoff RM (2018). A cluster-randomized trial of blood-pressure reduction in black barbershops. N E J Med.

[CR23] Voils CI, Maciejewski ML, Hoyle RH, Reeve BB, Gallagher P, Bryson CL, Yancy WS (2012). Initial validation of a self-report measure of the extent of and reasons for medication nonadherence. Med Care.

[CR24] Whelton PK, Carey RM, Aronow WS, Casey de Jr, Collins KJ, Dennison Himmelfarb C, DePalma S, Gidding S, Jamerson KA, Jones DW, MacLaughlin E, Muntner P, Ovbiagele B, Smith SC Jr, Spencer CC, Stafford RS, Taler SJ, Thomas RJ, Williams KA Sr, Williamson JD, Wright JT Jr (2018). 2017 ACC/AHA/AAPA/ABC/ACPM/AGS/APhA/ASH/ASPC/NMA/PCNA guideline for the prevention, detection, evaluation, and management of high blood pressure in adults: executive summary: a report of the American College of Cardiology/American Heart Association Task Force on clinical practice guidelines. Hypertension..

[CR25] Xu W, Goldberg SI, Shubina M, Turchin A (2015). Optimal systolic blood pressure target, time to intensification, and time to follow-up in treatment of hypertension: population based retrospective cohort study. BMJ.

